# Supplementary material for: Are carbon emissions trading and green financial instruments synergistic? -Comprehensive quantitative research based on content analysis
Source: PLoS One. 2024 Mar 7;19(3):e0298601. doi: 10.1371/journal.pone.0298601 (PMC10919601; doi:10.1371/journal.pone.0298601)
Supplement: S1 Data — (ZIP) [file pone.0298601.s001.zip › Data/地级市Z_F工作报告.zip!/╜¡╦╒/╦▐╟¿╩╨/└┤╫╘╣1⁄2╓┌║┼í╛┬φ┐╦╩2╛▌═°í┐_2010.docx]

各位代表：

现在，我代表市人民政府向大会报告工作，请予审议，并请市政协各位委员和其他列席同志提出意见。

刚刚过去的一年，是“十一五”的最后一年，也是宿迁发展历史上极不寻常、弥足珍贵的一年。在省委、省政府和市委的正确领导下，我们坚持以科学发展观为指导，紧紧团结和依靠全市人民，积极适应转变发展方式的新要求，成功应对金融危机带来的新挑战，努力克服经济社会生活中出现的新困难，顺利完成了市三届人大三次会议确定的各项任务，在“全面奔小康、建设新宿迁”的征程上迈出了坚实一步，为“十一五”发展画上了圆满句号。

——综合实力迈上新的平台。2010年，预计实现地区生产总值1015亿元，是“十五”末的1.9倍；人均地区生产总值达到21525元，折合3200美元。财政总收入五年翻三番，达到205.8亿元，年均增长51.4%；一般预算收入89.6亿元，在全国地级市排名上升到70位左右。全社会固定资产投资1010亿元，是“十五”末的3.8倍，年均增长30.3%。完成进出口总额11亿美元，其中出口9亿美元，分别是“十五”末的7.2倍和6.6倍。

——产业发展呈现良好态势。2010年，三次产业比例达到17.9：47.5：34.6，二、三产占比分别比“十五”末提高5.1个和3个百分点。酿酒食品、纺织服装、林木加工、机械电子等传统产业达百亿级规模，新兴产业重点企业达到103家。高效农业、高效渔业占比分别达到42.2%和53.6%。服务业增加值增速连续两年超过地区生产总值1个百分点以上。

——城乡面貌发生显著变化。到2010年底，全市城市化率达到41%，比“十五”末提高9.5个百分点。交通、水务、电力、通信等基础设施建设不断加强。市区建成区面积扩大到65平方公里，人口突破60万，相当于五年再建一个新城区。三个县城初步达到中等城市规模，沭阳县城人口超过50万。小城镇配套集聚功能明显增强，农民集中居住区累计新建住房12.8万套。

——民计民生得到持续改善。各级财政用于民生的投入累计达到289.7亿元，年均增长32.8%。2010年，预计城镇居民人均可支配收入、农民人均纯收入分别达到12740元、6875元，连续七年保持两位数增长。教育、医疗、科技、文化、体育等社会事业持续健康发展，社会保障制度实现全面覆盖，14项小康指标提前达到省定标准。

过去的五年，是我们坚定不移实施工业强市战略，大力培植新的增长点，经济始终保持高速增长的五年。财政一般预算收入、规模以上工业增加值、固定资产投资、社会消费品零售总额、进出口总额、农民人均纯收入等主要经济指标增速持续走在全省前列。坚持年年都是工业突破年、招商引资年、项目推进年，积极采取措施帮助企业应对国际金融危机影响，努力在外引内培中推动工业经济转型升级，加快新型工业化步伐。五年累计完成工业固定资产投资1700亿元，是“十五”时期的7.5倍。2010年，全市规模以上企业达到2476家，其中销售超亿元的157家，分别是“十五”末的3.9倍和6.8倍，苏酒集团成为全市第一家销售过百亿的企业；规模以上工业实现增加值284亿元，是“十五”末的2.8倍，增速连续六年位居全省第一。把开发区作为工业经济的主战场，以共建园区为载体深化与苏州的挂钩合作，各县区开发区全部获批为省级开发区，与苏州合作共建工业园区6家。2010年，全市开发区共实现业务总收入775.4亿元、财政总收入78.2亿元，分别是“十五”末的9.5倍和18.1倍。多渠道增加农业投入，大力推进农业产业化经营，五年累计新发展高效农业176万亩、高效渔业49.7万亩，152家市级以上龙头企业2010年合计实现销售收入134.5亿元。加强商贸流通、现代物流、休闲旅游等服务业集聚区建设，先后建成义乌国际商贸城、淮海建材城、海宁皮革城、国际汽配城、红利来建材大市场等专业市场。创建4A级旅游景区2家，获批为“中国优秀旅游城市”。2010年，全市共完成社会消费品零售总额286亿元，增长18.9%。引导江苏银行、交通银行、苏南五家农商行、紫金保险、省再担保公司等金融企业到宿迁设立分支机构，农村小额贷款公司总数位居苏北第一，在全省率先实现村镇银行县域全覆盖，成功当选“中国金融生态市”。截至2010年底，全市各类金融机构总数达到21家，是“十五”末的2.1倍，各项贷款当年净增202.2亿元，增长42.9%。

过去的五年，是我们全面加强基础设施建设，科学有序推进城乡一体化，区域综合承载能力显著提高的五年。强化规划的引领和控制作用，对中心城市重大规划方案实行国际征集，完成新一轮城市总体规划修编，实现城乡规划的全面覆盖。集中力量加快中心城市和县城建设，加强交通、供水、供电、商贸服务、环境保护等公共基础设施配套，完善和提升城市功能，并出台政策引导农村和外来人口加快向城市集聚。对幸福路、徐淮路、黄运路等商业街区实施改造提升，古黄河滨水核心区、霸王举鼎商圈等城市综合体初现雏形，“一湖两河”水系沟通、省第七届园博会园博园等景观和配套工程让滨水城市的特色日益凸显，中心城市整体形象发生重大变化。沭阳、泗阳、泗洪三个县城全面拓展了新的空间，配套建设了一批重要的公共基础和商贸服务设施，形成了自身的特色形象。稳步实施小城镇“1+4”工程，累计新增新型农贸市场150万平方米、商业街区100万平方米、居住小区250万平方米。启动建设农民集中居住区798个。徐宿淮高速、宿邳一级公路、市区南二环等高等级公路建成通车，京杭运河泗阳、刘老涧、皂河三座船闸建成通航，宿淮铁路、宿新高速、205国道、245省道、325省道等重点交通工程任务过半，新改建农村道路3816公里，所有行政村全部贯通水泥路，全市公路总里程突破1万公里，其中高速公路207公里。实施南水北调截污导流工程，新改扩建污水处理厂14座，铺设配套污水管网1109公里，全市污水日处理能力达到35.5万吨，超额完成“十一五”主要污染物减排任务。新沂河整治、淮北大堤加固、中心城市第二水源地等重点水务工程全面完成。电网最大网供负荷由2005年的53.5万千瓦增加到2010年的130.7万千瓦。完善城市长效管理机制，荣获“中国城市管理进步奖”。大力开展以“六清六建”、“三清一绿”为内容的农村环境综合整治，累计疏浚县乡河道4859公里、整治河塘1.1万面，完成改厕17.1万户，解决了137.6万农村人口饮水安全问题。持续推进城乡绿化，全市森林覆盖率五年提高7个百分点，城市绿化覆盖率、绿地率分别提高2.52个和2.77个百分点，人均公共绿地面积净增2.34平方米，成功创建为“国家园林城市”。

过去的五年，是我们不断深化改革创新，着力构建更加灵活的体制机制，内生发展活力明显增强的五年。顺利完成了市、县区政府机构改革，率先在全省开展乡镇机构改革试点，稳步推进事业单位改革。认真落实省直管县财政管理体制，授予宿豫区、宿城区、宿迁经济开发区、市湖滨新城必要的经济社会管理权限。大力推进行政审批“三集中、三到位”改革，行政审批事项和收费项目分别由2005年的587项和281项减少到2010年的331项和126项，实现行政权力全流程网上公开透明运行。不断加大财税改革力度，调整完善市区财政体制，实行招投标管理体制和国库集中支付改革。加大金融改革创新力度，新成立注册资本亿元以上担保机构15家，市、县农村信用社全部改制为农合行或农商行。洋河股份、秀强股份成功上市。深化国有企业产权制度改革，推动洋河、双沟通过股权收购组建苏酒集团。积极创新土地利用方式，推进城乡建设用地增减挂钩，实施“万顷良田”建设工程，促进城乡土地资源的科学配置和高效利用，五年累计盘活存量土地2.9万亩。完善以奖代投机制，成立市、县区教育、医疗卫生资产投资管理中心，有效解决对民办学校和民营医院的投入问题，促进各类教育卫生机构持续健康发展。

过去的五年，是我们不遗余力促进富民增收，逐步完善社会保障体系，人民群众得到实惠最多的五年。始终把创业作为发展之源、富民之本，在全社会大力弘扬创业文化，落实引导扶持政策，激发全民创业热情，获得“中国创业之城”称号。截至2010年底，全市个体工商户、私营企业分别达到16.2万户和2.9万家，比“十五”末净增7.8万户和1.56万家。加强公共就业服务体系建设，努力促进和帮助城乡各类群体就业，五年共新增城镇就业人口15万人，新转移农村劳动力50万人，城镇登记失业率控制在3.5%以内。着力构建广覆盖、保基本、多层次、可持续的社会保障安全网，2010年，城镇养老、医疗、失业、工伤、生育五项保险参保人数分别达到26.6万人、39.1万人、26万人、26.7万人和19.9万人，新农保参保人数达到180万人，城乡居民医保参保率均稳定在98%以上，实现了市域范围医保“一卡通”。以危旧片区改造为抓手，强化住房保障，五年累计新开工经济适用房21078套，新增廉租房2954套，建设拆迁安置房549万平方米，2010年当年实施危旧片区改造拆迁189万平方米，符合条件的城市低收入住房困难家庭全部纳入住房保障范围。以助困、助学、助老、助残为重点，努力解决弱势群体的实际困难。2010年，城乡低保标准提高到每人每月230元和155元，累计发放低保金2.65亿元、物价补贴3148万元；农村五保集中和分散供养标准提高到每人每年2900元和2300元，集中供养率提高到64%。持续实施脱贫攻坚工程，五年累计有88.6万农村贫困人口实现脱贫，特别是泗洪西南岗脱贫攻坚工作取得了关键性突破。

过去的五年，是我们协调发展各项社会事业，加强精神文明和民主法制建设，社会和谐程度稳步提升的五年。强化教育办学能力建设，推动各级各类教育实现快速协调发展。城乡义务教育全部免除学杂费。先后完成“六有”、“校校通”等教育工程建设，中小学校舍安全工程已完成加固5万平方米、新建103万平方米。2010年，全市3-5周岁幼儿入园率达到93.5%，初中毕业生升学率连续六年保持苏北第一，高考本科达线连续两年突破万人规模；宿迁学院本科在校生超过1.4万人；中职在校生增加到12.3万人，普职比调整到4.3:5.7；新增劳动力人均受教育年限提高到13年。科技进步贡献率达到41%。加强公共卫生及医疗服务体系建设，有效组织甲型H1N1流感、手足口病等医疗救治；市人民医院创成三级乙等医院，新建二级医院10所，完成108家乡镇卫生院建设任务，为848个村卫生室配置了基本设备；建立居民健康档案316万份。加强计生服务体系建设，新改建村级计生服务室1385个，县区及乡镇世代服务中心全部投入使用。连续举办创业文化节、西楚文化节，文学艺术创作取得丰硕成果，整合组建宿迁报业、广电两大集团，实现乡镇文化站全达标、农家书屋全覆盖、有线电视村村通。广泛开展全民健身活动，成功举办一系列大型体育赛事，参加第十七届省运会实现金牌、奖牌、总分全面翻番。反腐倡廉建设得到加强，政风行风有了明显改善。扎实推进平安宿迁、法治宿迁建设，建立健全社会矛盾预防化解机制，扩大法律援助范围，社会治安满意率持续位居苏北第一。不断完善应急管理机制，强化食品药品安全监管，安全生产事故起数、死亡人数连续七年“双下降”。成功创建“全国双拥模范城”。提前完成支援汶川地震灾区灾后重建任务，得到各方高度评价。统计、审计、档案、物价、气象、工会、妇女、青少年、工商联、人民防空、民族宗教、外事侨务、对台事务、无线电管理等事业都取得了新的进步，为全市经济社会发展作出了重要贡献。

各位代表，宿迁的发展始终得到了省委、省政府的高度重视和大力支持，特别是去年11月专门研究出台了支持宿迁发展实现更大突破的政策意见，为我们指明了前进方向、增添了强大动力、提供了有力支撑，推动宿迁不断实现科学发展新跨越。在这里，我代表市人民政府，向在各条战线上付出辛勤劳动的全市人民，向监督支持政府工作的人大代表、政协委员以及各民主党派、工商联、群众团体和离退休老同志，向为宿迁改革发展稳定作出积极贡献的中央和省驻宿单位以及人民解放军、武警官兵和人民警察，向直接参与宿迁建设的海内外客商、省级机关和苏州市广大援宿干部，以及所有关心支持宿迁发展的各级领导、各界人士，致以崇高的敬意和衷心的感谢！

当然，在总结成绩的同时，我们也清醒地看到，全市经济社会生活中还存在一些不容忽视的问题。特别是经济总量小、均量低的状况还没有根本改变；经济结构不尽合理，产业层次依然不高，新兴产业刚刚起步，现代服务业发展相对滞后，自主创新能力亟待提升；中心城市辐射带动功能仍需进一步增强，统筹城乡一体化发展还需迈出更大步伐；社会保障标准相对较低，一些群众生活比较困难，改善民生任重道远；影响社会和谐稳定的因素仍然较多。同时，少数部门的服务意识有待进一步增强，办事效率有待进一步提高，形式主义、官僚主义和消极腐败现象在少数人身上仍不同程度地存在。对这些问题，我们将高度重视，并在今后的工作中切实加以解决。

各位代表，今后五年是宿迁建成全面小康的关键阶段，也是实现更大突破的重要战略机遇期。根据市委关于制定国民经济和社会发展第十二个五年规划的建议，市政府制定了“十二五”规划纲要（草案），提请本次大会审议。按照纲要（草案），“十二五”时期全市经济社会发展的指导思想是：高举中国特色社会主义伟大旗帜，坚持以邓小平理论和“三个代表”重要思想为指导，深入贯彻落实科学发展观，以推动科学发展、谋求更大突破为主题，以创新发展方式、加速产业集聚为主线，以区域协调发展综合配套改革为动力，以保障和改善民生为根本，大力实施产业强市、城乡统筹、外向带动、创业富民、科教优先、生态立市六大发展战略，全面推进经济、政治、文化、社会以及生态文明建设，不断促进发展方式转变、经济体制转轨、社会结构转型，着力增强区域综合竞争力，确保如期实现全面小康，加快建成长三角地区新兴工商城市、绿色生态城市和创新创业城市，努力开创宿迁又好又快发展新局面。主要奋斗目标是，到2015年，完成“两大任务”：一是人均地区生产总值超过全国平均水平，二是确保如期实现全面小康目标。具体分为“两步走”：第一步，到2012年，打好发展基础。人均地区生产总值达到26400元左右，超过2009年的全国平均水平，年均增长14.8%；财政一般预算收入达到140亿元左右，年均增长25%；城镇居民人均可支配收入、农民人均纯收入分别达到16500元和8700元左右。第二步，到2015年，实现全面小康。人均地区生产总值达到42000元左右，超过全国平均水平，年均增长14.8%；财政一般预算收入达到270亿元左右，年均增长25%；城镇居民人均可支配收入、农民人均纯收入分别达到24500元和12700元左右。

提出这样的目标任务，既是省委、省政府和全市人民对我们的殷切期望，更是宿迁全面达小康、实现新突破的必然选择。展望新五年的发展，仍然是机遇与挑战并存，但机遇大于挑战。从宏观环境看，世界经济结构进入调整期，经济治理机制导入变革期，科技创新和产业转型处于孕育期，我国经济发展仍处于重要的战略机遇期。从宿迁自身看，人均地区生产总值已经突破3000美元，加快发展的基础更加坚实，正在进入经济稳定增长的新阶段，加上江苏沿海开发、长三角区域经济一体化的辐射带动，以及省委、省政府优惠扶持政策的支持拉动，一个梦寐以求的新的黄金发展期已经到来。因此，尽管国内外经济环境仍存在一些不确定、不稳定因素，发展过程中也还面临着资源约束、要素瓶颈等各种压力，但只要我们抓紧抓牢大好机遇，充分利用各种有利条件，未来五年的发展潜力巨大，前景广阔，大有作为。

在具体工作中，我们将围绕“十二五”规划纲要确立的目标任务，集中力量实施好六大发展战略。立足“产业强市”，着力打造产业集聚新高地。加快产业转型升级，招大培强主导产业，发展壮大新兴产业，提高一产发展水平，拉动三产快速发展，实现经济总量快速扩张、发展质量明显提升。到2015年，全市三次产业比例达到9：52：39，培育形成新兴产业、酿酒食品、纺织服装三个千亿级和林木加工、机械电子、商贸物流三个五百亿级产业集群。着眼“城乡统筹”，着力构筑一体发展新格局。围绕城乡一体化、城市现代化，坚持以就业集中人口、以人口繁荣城市，着重推动中心城市和三个县城协调发展，加快建设现代化中等城市群；优先发展重点中心镇，稳步发展一般小城镇，合理建设农民集中居住区。在此基础上，科学配置和利用城乡要素资源，实现城乡互动发展、共同繁荣。到2015年，市区和沭阳县城人口均达80万以上，泗阳、泗洪县城达到50万以上，全市城市化率达到55%左右。强化“外向带动”，着力拓展经济发展新空间。把对外开放作为实现更大突破的强大引擎，加快开发区转型升级和升档进位，更加主动地对接沿海开发、融入长三角，全面提升外经外贸和利用外资水平，促使开放型经济在三年内实现倍增。到2015年，全市进出口总额达到40亿美元，实际到账外资7亿美元，年均增速双双达到30%。突出“创业富民”，着力拓宽群众增收新渠道。进一步扩大投资领域，优化创业环境，支持更多群众走上创业致富道路。深化分配制度改革，全力扩大就业再就业，加强社会保障和帮扶救助，促进城乡居民收入普遍较快增长，确保实现“五年倍增”目标。致力“科教优先”，着力增强创新发展新支撑。突出教育第一基础、人才第一资源、科技第一生产力的作用，加快推进教育现代化，多渠道增加科技投入，努力培养和引进更多留得住、用得上的人才。到2015年，全市教育发展主要指标在苏北领先；研发经费支出占地区生产总值的比重达到1.5%，高新技术产业产值占规模以上企业的比重提高到12%以上，科技进步对经济增长的贡献率达到47%；人才总量超过40万人。坚持“生态立市”，着力增创持续发展新优势。把绿色生态作为最稀缺的资源、最宝贵的财富，始终恪守环保优先方针，深入开展资源节约型、环境友好型社会建设，加快发展循环经济、绿色经济、低碳经济，把“生态宿迁、绿色家园”的金字招牌擦得更亮。到2015年，全市森林覆盖率达到30%，万元地区生产总值能耗和各类污染物排放总量确保完成省定任务。

各位代表，回首过去五年乃至建市以来的历程，宿迁始终因改革创新而发展，因改革创新而突破，因改革创新而辉煌。我们要担当起全面达小康、实现新突破的历史使命，必须紧紧抓住区域协调发展综合配套改革试点的历史机遇，以敢试敢闯的勇气破解发展难题，以敢为人先的精神谋求新的跨越，永葆改革创新的生机与活力，让改革创新的旗帜在宿迁大地永远飘扬，努力把“十二五”的宏伟蓝图变为美好的现实！

各位代表，2011年是“十二五”规划的开局之年，也是全面建成小康社会、实现宿迁发展更大突破的起步之年。做好今年各项工作，保持和巩固经济快速增长的良好势头，意义重大，影响深远。综合考虑各方面因素，今年全市经济发展的主要预期目标是：地区生产总值增长14%；财政一般预算收入增长25%；500万元以上固定资产投资、进出口总额、实际到账外资均增长30%；规模以上工业增加值增长20%以上；社会消费品零售总额增长18%；研发经费支出占地区生产总值比重达到0.8%；节能减排完成省定目标。具体抓好七个方面工作：

（一）坚持不懈引大培强，提升工业经济集聚发展水平。着力实施“五大计划”。一是亿元以上大项目推进计划。全年确保新引进、新开工、新竣工亿元以上项目300个、100个和100个，每个县区引进投资15亿元以上或6000万美元以上的大项目不少于2个，全市工业固定资产投资增长35%以上。二是重大项目竣工提速计划。重点帮助和推动长电科技、德力化纤、蒙牛乳业、达利食品、旭源科技、欧亚薄膜、荣马能源、常盈科技等在建重大项目尽快投产达效。三是新兴产业扩张计划。强化生产要素保障，加大政策支持力度，全年确保新开工战略性新兴产业项目30个，新能源、新材料、软件和服务外包产业年内销售收入均增长50%以上，高新技术产业产值占规模以上企业的比重提高到8%以上。四是传统产业转型升级计划。采取针对性帮扶措施，确保酿酒食品、纺织服装、林木加工、机械电子产业年内分别实现销售收入320亿元、200亿元、230亿元和240亿元。五是大企业大集团培育计划。继续实行一企一策、分类指导，全年确保新培育销售收入超2000万元的规模企业200家，新增销售收入超亿元的企业20家以上。加快园区转型升级步伐。坚持以特色园区建设引领产业集群发展，重点支持洋河双沟酿酒产业园、国家级食品产业园、双向拉伸薄膜产业园等特色园区建设，通过规划指导、政策引导和环境营造，吸引相关企业向园区集中，形成核心竞争优势，促进开发区升档进位。今年各省级开发区在全省综合排名确保上升5个位次，宿迁经济开发区力争年内建成国家级开发区，市软件与服务外包产业园全年确保新增入园企业30家以上、实现业务收入3亿元以上。推动南北共建园区的产业对接、项目对接，苏宿工业园区年内力争引进2个5亿元以上、3个亿元以上项目，其他共建园区确保分别引进亿元以上项目不少于5个。加强经济运行调节。根据财力增长相应增加工业引导资金投入，与省财政支持宿迁产业发展的专项资金统筹使用，通过集中投入、重点投入、连续投入，确保政策和资金效益发挥最大化。科学搞好经济运行监测分析，及时掌握重点企业、行业的经营状况和发展态势，针对性做好煤电油气等生产要素的供给保障。认真落实减税减费政策，及时兑现奖励扶助资金，千方百计帮助企业降本增效。

（二）大力发展农业产业化经营，加快推进现代农业建设。加强现代农业产业园区建设，引导农业适度规模经营。在完成首批市级园区认定的同时，重点做好4个省级农业产业园和1个省级渔业产业园申报认定工作。全年确保新增高效农业40万亩、高效渔业15万亩，其中新发展设施农业15万亩，新增适度规模经营面积45万亩。加大农业新品种、新技术、新模式的引进、示范和推广力度，着力提高农产品质量和效益。大力实施以籼稻改粳稻、旱田改水田、直播改栽插、手栽改机插为主要内容的水稻“四改”工程，加快提升粮食综合生产能力。加强蔬菜种苗繁育中心建设，年内全市蔬菜集中育苗能力达到3亿株以上。积极发展生态农业、循环农业，推行无害化、标准化生产，健全完善质量检测网络，支持农产品品牌创建，年内确保新创建地理标志商标1件、省级著名商标10件、省级名牌农产品10个，新增绿色食品20个、有机农产品10个。加强农产品加工集中区建设，带动农业龙头企业发展。年内各县区确保规划和启动建设年产值15亿元以上的农产品加工集中区1个，全市新认定市级以上农业龙头企业20个，其中国家级1个、省级2个。着力推进农民合作经济组织转型升级，全年分别新发展省、市级“五好”示范社10个和30个。健全完善农产品市场流通体系，市区华东农业大市场一期工程确保上半年投入运营，各县区也加快建设区域性的特色农产品交易市场。大力推进农业基础建设，加强农业综合开发。特别是抓住中央重点支持农村中小型水利工程建设的机遇，采取试点推动、示范带动的方式，统筹推进水利合作组织建设，加快灌区改造、小型农田水利、农村河道疏浚等农田水利工程建设，集中整治245省道、新宿邳路、新宿沭路沿线以及西南岗农田沟渠，改善农田排灌条件，建设一批旱涝保收、高产稳产高标准基本农田，全年确保新建成高标准农田20万亩。认真落实购机补贴政策，鼓励农业企业、生产大户添置农机设备。不断提高高效农业政策性保险参保率，切实增强农业抗风险能力。

（三）推动服务业提速增效，积极引导和扩大消费需求。大力实施服务业提速计划，改造提升传统商贸流通业，加快发展生产性服务业，提高现代服务业发展水平。重点打造核心商圈、总部经济、商务楼宇和特色街区，全面提升城区商贸质态。构筑完善现代物流体系。市区依托在建的宿淮铁路和京杭运河宿迁中心港，重点推进宿迁中心港物流园区、江苏中联物流中心、宿迁翔盛物流中心、宿迁交通物流集装箱站场建设。各县区年内也相应建设一批功能完备的物流基地和物流中心，培育壮大一批物流企业。高度重视旅游业发展，依托生态资源优势，打造华东地区知名的生态旅游目的地。整合骆马湖周边旅游资源，加快运河文化城、中国水城、温泉度假城等重大旅游项目建设，启动骆马湖水上旅游开发，打造皂河旅游古镇，确保年内骆马湖省级旅游度假区创建成功。推进以洪泽湖湿地公园为重点的生态旅游示范区建设，重点开发特色旅游乡镇（村）、生态休闲农庄、特色农业观光园、民俗风情园、农家乐等乡村旅游产品，年内确保创建旅游特色村9家。积极开展“文化产业发展年”活动，加快泽达创意产业园、苏酒博物馆、市图书馆新馆、新华书城等重大文化项目建设，精心筹拍一批特色影视作品，促进报业集团、广电集团、楚凤文化等重点文化企业做大做强。努力搞活流通促进消费。有效落实家电、汽车、摩托车下乡和以旧换新等鼓励消费的政策措施，巩固发展城乡消费持续增长的好势头。健全城乡商贸流通体系，支持纺织品、服装、家具、建材等专业市场建设，引导城市商业网点向农村延伸。积极发展新型消费业态，加快餐饮酒吧、休闲会所、教育培训、医疗保健等服务场所建设，培育汽车、旅游、住房等消费热点，促进市场持续繁荣。大力整顿和规范市场秩序，加强产品质量和价格监管，营造快捷便利、安全放心的消费环境。

（四）全力加快重点工程建设，集中展示城市整体形象。紧扣时间节点，倒逼推进园博园及相关配套服务设施建设，高质量承办好省第七届园博会。上半年园博会主场馆力争竣工并启动布展工作，博览园及罗曼园配套设施和景观工程全面完成；八月份完成宿迁景区建设任务；九月份星辰国际会议中心竣工运营，年底前城市展览馆主体工程基本完工，运河国际会议中心完成内外装修。着力打造市区核心地标群。年内宝龙城市广场商业部分建成运营，金鹰天地广场进入中心店主体施工，宿迁1897、中央国际购物广场三季度开工建设，加快中豪国际商业中心、金三角金融财富广场、恒力五星级酒店等项目建设进度。加强市区应急避难场所建设，科学开发利用城市地下空间。加快110指挥中心、公交调度指挥中心建设，新区商务楼、便民服务中心以及江苏银行、民丰银行综合办公楼年内确保投入使用。快速推进沭阳南部新城、泗阳运河新城、泗洪中心商务区建设，不断增强县城的集聚能力和带动功能。大力开展“精品城市”创建。规范户外广告牌匾设置，加快市区路灯亮化集中监控系统二期工程建设，大力整治沿路、沿街的残破危旧建筑，全面修复损坏的人行道板等公共设施；集中力量整治背街小巷、河道沟边卫生死角，对城区主次干道、公共场所实行全天候保洁；加强建筑施工和道路扬尘整治，严控餐饮附属设施油烟排放，保持城市空气质量良好。深入开展“绿化品位提升”行动，加快建设城市大型绿廊和干道绿色通道，重点打造市府新区、宿豫新区、宿城新区三大园林景观精品片区和20个园林景观精品庭院、20条园林景观精品道路。始终坚持镇当城建的理念，按照中小城市发展要求，对省市重点中心镇进行全面规划建设，着重支持洋河镇发展，打造未来的洋河新城。按照“三集中、三靠近”的原则，尊重农民意愿，推进农民集中居住区与小城镇一体规划、建设和发展。同时，充分考虑城市化快速推进所带来的公共需求变化，及时调整优化教育、卫生等公共服务资源布局，积极鼓励有条件、有实力、有能力的农民到中心城市、县城和重点中心镇购房居住、就业生活，不断加快城市、城镇人口集聚的步伐。

（五）全面完善基础设施支撑体系，提高区域综合承载能力。重点推进四个方面的基础工程建设。一是重大交通工程。全力加快宿新高速宿迁段、205国道、344省道、325省道以及发展大道南延等工程建设步伐，326省道沭阳段、121省道宿迁段、245省道泗洪至安徽段确保年内启动，京杭运河宿迁一号桥确保年内建成通车；宿淮铁路宿迁段年内建成，并认真做好徐宿淮铁路前期和启动工作；京杭运河宿迁中心港、成子河船闸工程确保年内开工。二是城市水务工程。重点推进古黄河雄壮河湾景区改造、环城西路以西景观提升以及民便河景区改造、七堡引湖枢纽等工程，古黄河与民便河内环沟通工程确保年内开工建设；加快西民便河综合整治和管网清淤，六塘河疏浚整治工程确保三季度完工，市区积水地段改造、古黄河疏浚整治、城区排涝泵站和排水管网工程年内完成，市第二水厂年底前实现并网供水。三是节能环保工程。确保完成既有建筑节能改造2.5万平方米，光大垃圾焚烧发电厂、市危险废弃物填埋场、苏华达新材料脱硫、河西污水处理厂二期以及相关污水处理厂强化脱氮等工程年内建成运行。切实做好新沂河北偏泓尾水通道扩容工程前期工作，按计划推进南水北调截污导流扩容工程建设，确保重点考核断面水质达标率明显提升。四是区域电网工程。大力完善高压电网，强化中低压电网，推动国电二期2×600兆瓦发电工程尽快启动建设，年内确保500千伏双泗变扩建和4个220千伏输变电工程按期达标投产，切实提高配电线路联络率、配网供电能力及供电可靠性。同时，继续加大供气、供热以及广电管网、加油站等基础设施投入力度，全面提高保障水平和承载能力。

（六）着力深化综合配套改革，更大力度扩大对外开放。不断完善行政管理体制改革，在巩固市、县区政府机构改革成果的基础上，加快实施洋河镇行政管理体制改革试点，积极开展市级扩权强镇试点，激发镇域经济发展活力。大力推进招投标管理创新，积极探索公共资源交易管理新机制。不断强化财税体制创新，完善市区财税管理体制，加大预算资金统筹调配力度，稳妥实施事业单位绩效工资制度。继续争取市外股份制银行来宿设立分支机构，支持具备条件的农合行改制为农商行，推进新型农村金融组织试点。积极引进风险投资基金、股权投资基金，鼓励和支持企业通过上市、发行债券等手段进行直接融资，探索以政府主导、社会参与的方式设立创业投资公司，年内各县区分别设立1家注册资本1亿元以上的创投公司。稳步推进城乡建设用地增减挂钩和万顷良田建设工程，年底前市区和三个县分别建成不少于1000公顷连片的高标准农田。积极探索在市域范围内综合统筹和合理配置土地资源的办法，创新土地流转和土地股份合作方式，研究农村集体非农建设用地进入市场的有效途径，开展土地承包经营权置换城镇社会保障、农村宅基地使用权置换城镇保障住房的“双置换”试点。坚持以改革促开放，切实提升经济国际化水平。利用海关运行的有利条件，大力发展加工贸易，培育外贸主体，加快出口加工区和保税物流园区创建，争取宿迁国检上半年获批。支持企业参加境内外展会开拓国际市场，确保全年实现进出口总额14.3亿美元。加大外资引进力度，全年新批外商投资企业70家，实际到账外资2.4亿美元。积极鼓励企业“走出去”到境外投资，拓展对外劳务合作和境外建设市场，年内自营外派劳务600人，实现营业额2000万美元以上。

（七）切实加强自主创新能力建设，推动创新型经济快速发展。全面落实与省科技厅的会商制度，抓好科技园区和创新平台建设，努力把宿迁经济开发区科技产业园和市软件与服务外包产业园建成一流的省级现代科技园区。围绕优势主导产业和特色园区发展，加快产业研究院建设步伐，支持智能输变电设备、功能膜材料、生物酿酒、木材加工等研究院建设，确保国家白酒产品质量监督检验中心年内建成使用，国家玻璃制品质量监督检验中心力争年内立项建设。积极探索政产学研合作长效机制，精心筹办好“中国宿迁生态科技博览会暨产学研合作洽谈会”，推动企业与重点高校、科研院所开展多种形式联合，吸引高水平的专业团队参与产业研究院建设，争取更多的高新技术项目和产学研基地、研发机构落户宿迁，年内确保实施合作项目200个以上。加强科技企业孵化器、科技创业服务中心建设，切实完善和提升服务功能。继续加大对企业技术中心、工程中心等研发机构的扶持力度，引导企业增加研发投入，确保年内研发经费支出占地区生产总值比重比上年提高0.2个百分点，50%的规模以上工业企业建有研发机构，省级工程技术研究中心总数达到35家以上。支持企业参与高新技术认定，年内全市按国家新标准认定的高新技术企业总数达到30家以上。加强知识产权保护，鼓励发明创造，全年专利申请量确保超过1000件。广泛开展名牌商标和名牌产品创建活动，全年创建中国驰名商标2件以上、省名牌产品20个以上。足额安排人才发展专项资金，全面启动“十大重点人才工程”，统筹实施好领军人才“双百计划”、产学研人才“双千计划”、新兴产业人才“双万计划”等重大人才计划，年内确保引进国内外高层次科技人才100名，培训科技企业家100名，签约外协专家200名以上。认真实施高技能人才培养“金蓝领”计划，拓展行业职业技能鉴定，全年确保培养技师、高级技师600人，组织行业鉴定2000人。

各位代表，我们的政府是人民政府。让人民生活更加美好，是政府义不容辞的责任。围绕更大力度地保障和改善民生，今年将以就业创业、城乡安居、医疗教育、公交通达、扶贫济困、养老保障、便民服务、环境改善等八个方面的民生实事项目建设为重点，切实解决好与群众生活密切相关的实际问题。全市财政用于民生的投入确保增长30%以上，城镇居民人均可支配收入确保增长14%、农民人均纯收入增长13%，让人民群众通过实实在在的生活感受，进一步提高对政府工作的满意度。

千方百计推动创业扩大就业。制定更富针对性和可行性的政策措施，推动民营经济更好更快发展。完善创业配套服务，加快创业孵化和培训基地建设，扩大创业担保贷款规模，鼓励企业剥离非核心业务向创业者开放。全年确保发放青年创业小额贷款5000万元，新增个体工商户3万户、私营企业3000家。大力开展各类培训，全年完成农村劳动力转移及实用技术培训24.2万人、农民创业及“致福工程”培训1.8万人，对2000名退役士兵和复员士官开展免费中高级职业技能培训。进一步增加就业再就业资金安排，健全完善四级联网、资源共享的公共就业服务体系，多渠道开发就业岗位，加大就业援助力度，促进各类群体充分就业，动态消除城镇零就业、农村零转移家庭。全年确保城镇新增就业2.5万人、农村劳动力新增转移就业4.5万人，帮助8500名失业人员实现再就业，新建大学生见习基地72个，城镇登记失业率控制在3.5%以内。在住房、子女入学、社会保障等方面采取更有针对性的措施，为本地劳动力就地就近就业、外出务工人员返乡返土就业、市外人员选择来宿迁就业创造更好的条件。强化劳动监察和劳动争议调解仲裁，适时提高最低工资标准，引导企业逐步提高职工劳动报酬，不断改善职工的福利待遇。

全面提高社会保障水平。大力宣传贯彻《社会保险法》。不断完善保障体系，提高保障标准，努力实现人人享有社会保障的目标。继续扩大各类社会保险覆盖面，积极实施工伤保险市级统筹，稳步提升保险统筹层次，全市五险扩面新增合计18万人。将未参保集体企业退休城镇户籍人员全部纳入基本养老保险范围，新征地农民社会保障实行“即征即保”，新农保基础养老金发放率达到95%以上，城镇居民医保及新农合参保率都稳定在98%以上。落实城乡低保和农村五保标准自然增长机制，年内城乡低保标准分别提高到240元、210元，农村五保集中和分散供养标准分别提高到3200元、2600元，集中供养率达65%以上。深入开展城乡医疗救助，加快同步结算平台建设，实现医疗救助与城镇居民医保、新农合的无缝对接，对获得医保补偿以后的个人自付部分再给予不低于30%补助，为贫困家庭儿童重大疾病提供特殊救助。建立健全城乡基本养老服务体系，大力实施农村敬老院“关爱工程”，加快居家养老服务中心建设，全年完成“关爱工程”建设面积2.6万平方米，新增床位510张，新建居家养老服务中心220个。扎实推进新一轮全国双拥模范城（县、区）创建，认真落实好各项优抚安置政策。

深入实施扶贫济困工程。不断壮大村级集体经济，增加贫困户收入，年内确保254个省定经济薄弱村集体经济收入全部达到5万元以上，新建成脱贫目标实现村70个，年收入低于2500元的贫困户全部实现脱贫。支持企业直接参与结对帮扶村建设，允许农民和村级组织以非农建设用地资源和资金投入等形式参股土地开发，鼓励省定经济薄弱村利用各类扶贫资金，到乡镇工业集中区或县区开发区建设标准厂房进行出租，有效增加集体收入。加大财政投入，强化扶贫小额贷款支持和贫困劳动力培训，年内确保发放贷款5亿元以上、培训2万人以上。全面加快泗洪西南岗地区脱贫富民步伐，确保有劳动能力的贫困人口年内全部脱贫，提前一年完成脱贫攻坚任务。高度重视发展慈善公益事业，加大帮困济贫、助残扶弱力度，让各类特殊社会群体得到更多关爱。年内为1000名6岁以下残疾儿童实施免费康复训练，为2000名贫困残疾人家庭、2000例白内障致盲残疾人分别实施无障碍改造及辅助器具适配和免费复明手术。

切实提升公共服务能力。注重教育资源的合理配置与优化布局，不断缩小区域、城乡、学校之间差距，推进教育优质均衡发展。深入实施教育现代化建设，泗洪县、宿城区确保年内通过省评估验收。扎实推进校舍安全工程建设，全年确保完成78.8万平方米加固改造、57万平方米拆除新建任务。全面提升基础教育发展水平，积极培育壮大优质教育资源，提升普通高中办学内涵，年内确保60%以上的普通高中达到省优质标准。继续大力发展职业教育，支持宿迁职业技术学院、泽达职业技术学院、淮海技师学院、宿迁经贸学院、宿迁技师学院等院校做大做强，推动职业院校品牌化发展，江苏农民培训学院一期工程确保上半年投入使用。全面落实“省市共建”措施，争取宿迁学院建成独立设置的本科院校。旗帜鲜明地支持民办教育发展，鼓励民办学校通过办学权转让、融资扩股、校企联办、组建集团等方式调整重组、增强实力。加强教育救助工作，对困难家庭幼儿接受学前教育费用予以减免，帮助200名家庭贫困、品德优秀的应届普通高中学生圆梦大学，积极筹措资金援助希望小学2所。结合宿迁实际落实好基本药物制度，在全市所有乡镇医院全部使用基本药物目录内药物，降低药品价格，提高报销比例。认真实施好基本公共卫生服务项目，促进公共卫生服务逐步均等化。加强各级卫生医疗机构建设，年内完成4家县级医院、5家精神病院、21家中心乡镇卫生院（医院）基础设施改扩建工作，确保市人民医院新病房楼投入使用，市东方医院门诊楼主体完工，市妇产医院、佳宝儿童医院按三级专科医院标准启动新建。继续通过以奖代投方式，加大政府对医疗卫生事业投入。大力实施“文化惠民”工程，加快完善基层公共文化设施，广泛开展文化进社区活动，鼓励扶持民间文艺团体健康发展，有线电视“进村入户”新发展农村用户10万户。积极探索创建生态体育试点市，加快体育设施场馆建设，打造市区“十分钟健身圈”和“古黄河生态体育万米健身带”，市全民健身中心确保年内投入使用，市体育中心主体完工，筹备组织好市第三届运动会。实施人口计生服务体系提质工程，完善县乡世代服务中心功能，全年人口自然增长率控制在5‰以内，计划生育率保持在95%以上。

努力改善群众居住生活环境。重点实施好四项工程。一是城乡安居工程。在切实增加普通优质商品房供给的同时，按照“产权置换、就地就近”的原则，加快城市危旧片区改造，增加保障性住房供应，使各类社会群体的居住条件得到不同程度的改善。全市年内新增廉租房、公租房3430套、经济适用房5210套，完成危旧片区改造拆迁150万平方米，新建拆迁安置房200万平方米，所有开发区建成1个面积不低于5万平方米的蓝领服务中心。农民集中居住区新建住房2.5万套。二是公交通达工程。加快公交一体化改造步伐，全市新增城乡公交线路10条，新投放公交车100辆、更新50辆；市区开通4条夜班线路，改造公交候车亭133座；新建5个农村客运站、150个农村客运候车亭，年内85%以上的行政村开通公交线路；加大农村公路和桥梁改造力度，年内完成207公里农村公路和30座桥梁改造任务。三是便民服务工程。加强小区物业管理，提高物业管理水平。按照“管理有序、服务完善、文明祥和”的建设标准，加强城乡社区服务设施建设，全市60%的村居、50%的乡镇年内都相应建立功能配套的社区综合服务中心；加快村级通邮站建设，为农民提供用邮、缴费、农资销售等服务，年内确保有30%的行政村完成建设任务。四是环境整治工程。深入开展农村环境综合整治，加强水环境、农业面源污染、畜禽水产养殖污染以及生活垃圾的治理，年内新建户用沼气池3000个、畜禽养殖沼气治理工程10处、秸秆气化集中供气工程2处，完成改厕5.5万户。全年新造林4万亩，全市森林覆盖率保持在28%以上。

全力维护社会大局和谐稳定。切实加强安全生产工作，强化对重点行业和企业、交通、消防等安全隐患专项整治，继续降低事故总量和伤亡人数，减少职业危害，坚决防止重特大事故发生。高度重视群众来信来访，强化领导干部定期接访、主动下访，努力把问题解决在基层、化解在萌芽状态。全面启动“六五普法”，切实完善人民调解、行政调解、司法调解三位一体的大调解机制，建立调处化解矛盾纠纷综合平台，加强法律服务和援助工作，帮助群众通过正当渠道反映和解决个人诉求。深入推进平安宿迁、法治宿迁创建，加强社会治安综合治理，加快社区（居委会）综治办、警务室建设，健全完善道路监控网“320”工程等防控体系，严厉打击各种违法犯罪活动，不断增强人民群众安全感。深入开展全民国防教育，扎实做好民兵预备役和国防动员工作。大力整顿规范食品药品生产流通秩序，提高食品药品安全保障水平。全面加强社会管理创新，切实提高预防和处置突发公共事件能力，让广大人民群众在安定和谐的社会环境中生产生活。

各位代表，宿迁的发展已经站在了新的历史起点上。全面建成小康社会、实现发展更大突破，对政府工作提出了新的更高要求。各级政府必须进一步加强自身建设，切实树立勤勉尽责、务实清廉的良好形象，努力提高行政管理和服务水平。一是全面推进依法行政。严格按照法定权限和程序行使权力、履行责任。自觉接受人大及其常委会的法律监督和工作监督，接受政协民主监督，强化审计、监察等专门监督，支持新闻媒体的舆论监督。健全完善重大事项社会公示、听证和专家咨询制度，增强决策的透明度和公共参与度，提高政府决策的民主化和科学化水平。强化行政执法监督，全面推行执法责任制、执法公示制和评议考核制，完善行政复议制度和过错追究制度，切实规范执法行为。认真办理人代会议案、人大代表建议和政协委员提案，确保100%答复、100%满意。二是加强和改进政风行风建设。不断完善行政审批“三集中、三到位”改革，推动行政权力全部上网公开透明运行。切实精简会议、减少文件，努力提高政府的办事效率和服务水平。加强政务公开和政府信息公开，健全完善新闻发布制度，充分发挥“网络问政”平台的作用，不断扩大群众的知情权、参与权和监督权。加强对教育收费、医疗服务、师德医德以及土地征用、物价、拆迁等热点行业、重点部门和关键岗位的监督管理，坚决防止和制止损害群众利益的不正之风。三是深入开展廉政建设和反腐败斗争。坚持不懈地加强理想信念和廉洁从政教育，自觉做到一身正气、干净干事。坚持用制度管权、按制度办事、靠制度管人，从源头上遏制腐败现象的滋生。严格规范建设工程招投标、政府采购、土地出让等公共资源管理，严肃查处各类违法违纪行为。大力发扬勤俭节约、艰苦奋斗的优良作风，着力加强公务接待和公务用车管理，坚决取消一切不合理、不必要的经费开支，把有限的资金更多地用于改善民生和公共服务，努力营造风清气正、廉洁高效的政务环境。

各位代表，回顾“十一五”，辉煌成就鼓舞人心；展望“十二五”，宏伟蓝图催人奋进。让我们在市委的坚强领导下，抢抓机遇，乘势而上，继往开来，奋发图强，全力开创宿迁科学发展、跨越发展的新局面，为全面达小康、实现新突破，谱写宿迁人民更加美好生活的新篇章而努力奋斗！
